# Predicting the SARS-CoV-2 effective reproduction number using bulk contact data from mobile phones

**DOI:** 10.1073/pnas.2026731118

**Published:** 2021-07-14

**Authors:** Sten Rüdiger, Stefan Konigorski, Alexander Rakowski, Jonathan Antonio Edelman, Detlef Zernick, Alexander Thieme, Christoph Lippert

**Affiliations:** ^a^Machine Leaning Unit, Department of Engineering, NET CHECK GmbH, 10829 Berlin, Germany;; ^b^Digital Health–Machine Learning, Hasso-Plattner-Institut, Universität Potsdam, 14482 Potsdam, Germany;; ^c^Hasso Plattner Institute for Digital Health, Icahn School of Medicine at Mount Sinai, New York, NY 10029;; ^d^Department of Radiation Oncology, Charité-Universitätsmedizin Berlin, 13353 Berlin, Germany;; ^e^Digital Clinician Scientist Program, Berlin Institute of Health (BIH), 10178 Berlin, Germany;; ^f^Department of Medicine, Stanford University, Stanford, CA 94305;; ^g^Department of Biomedical Data Science, Stanford University, Stanford, CA 94305

**Keywords:** COVID-19, network science, epidemiology

## Abstract

Over the last months, cases of SARS-CoV-2 surged repeatedly in many countries but could often be controlled with nonpharmaceutical interventions including social distancing. We analyzed deidentified Global Positioning System (GPS) tracking data from 1.15 to 1.4 million cell phones in Germany per day between March and November 2020 to identify encounters between individuals and statistically evaluate contact behavior. Using graph sampling theory, we estimated the contact index (CX), a metric for number and heterogeneity of contacts. We found that CX, and not the total number of contacts, is an accurate predictor for the effective reproduction number R derived from case numbers. A high correlation between CX and R recorded more than 2 wk later allows assessment of social behavior well before changes in case numbers become detectable. By construction, the CX quantifies the role of superspreading and permits assigning risks to specific contact behavior. We provide a critical CX value beyond which R is expected to rise above 1 and propose to use that value to leverage the social-distancing interventions for the coming months.

## Significance

Numerous COVID-19 studies used mobile phone data but with limited power in accounting for infection numbers. We have evaluated deidentified Global Positioning System (GPS) data from over 1 million devices in Germany and inferred contacts from coproximity of devices. Through calculating the contact graphs, we derived a contact index (CX) that exhibits a high correlation with the incidence-based reproduction number R so that changes in CX precede those in R by more than 2 wk. CX thus is an early indicator for outbreaks and can be used to guide social-distancing policies. Further questions, including those for the efficacy of vaccination, can be addressed with our method. We discuss limitations, e.g., transmission on international travel, and the relation to superspreading.

In December 2019, the novel coronavirus, SARS-CoV-2, caused a sustained pandemic with more than 140 million confirmed cases and more than 3 million deaths as of April 2021. SARS-CoV-2 is highly contagious with an estimated basic reproductive number $R_{0}$ between 1.5 and 4 (1–4). It may spread invisibly in the community until a local outbreak becomes noticeable by a larger number of severe clinical cases. Nonpharmaceutical interventions (NPIs) have been considered an important tool to contain the spread of the virus. In Germany, before the implementation of strict interventions, an exponential growth of case numbers was recorded with an effective reproduction number R of about 3. The R value characterizes the epidemic risk of a population and is defined as the average number of new infections caused by a single infected individual in the susceptible population. Changes in the temporal or effective R can occur for instance in dependence on immunization of individuals and on social-distancing measures. Thus, estimates of R provide an important measure of public health policy. One problem with the R value and its properties is the underlying mathematical model used to estimate it. In Germany, as in many other countries, the official R calculation is based on evolution of the number of newly infected individuals recorded by health authorities in Germany.

The problem with the model is this: Due to asymptomatic carriage of SARS-CoV-2 (5, 6), a large portion of infected individuals remain undetected. It was determined that 44% of secondary cases are infected through presymptomatic transmission events (7). With an estimated incubation time of 5 d (3), a reported time delay of 6 d between symptoms and diagnosis based on laboratory tests (8), and additional time delay for reporting to authorities (9), laboratory testing does not appear to be sufficient for early outbreak detection and outbreak control given the short infection doubling time of SARS-Cov-2 of 1.4 to 2.5 d (10).

In response to this problematic model, other methods must be considered that allow for the early detection and control of outbreaks. Soon after the beginning of the pandemic, various mathematical and epidemiological models have been used in attempts to explain or predict case number evolution (11–13). Additionally, multiple cell phone apps have been developed to monitor the health of the population or record contacts between users. Several solutions focused on determining contacts based on Bluetooth low energy (BLE) to inform users about contacts to infected individuals. It was often found that researchers need to gather a significant number of users to enable identification and interruption of infection chains (14) and in many countries the success of the apps has been disappointing (15). Also, privacy concerns have frequently been raised and many countries chose to limit the capabilities of the method so that the individuals’ privacy rights are not compromised (16).

A related approach, employed soon after the beginning of the pandemic, is that of analyzing bulk mobile phone data with location or proximity information but anonymized user identity (17). In contrast to contact-tracing apps, a blending of the mobility data with case data, which increases the risks of privacy breaches, is precluded. Yet, one hopes to explain and control the infection behavior of the population using a statistical description of the conditions in which the transmissions occur.

Most studies in this realm used aggregated and anonymized records from phone companies that quantified movements between regions. Publications therefore emphasized the advantage to assess efficacy of NPIs, for instance by measuring travel metrics, and to improve epidemiological models (18–20) (reviewed in ref. 17). Early studies indeed showed the relevance of mobility in the case of the spread of SARS-CoV-2 from Wuhan to other cities (21). However, the strong associations between mobility and case growth rates are absent during later phases of the epidemic, suggesting that mobility alone cannot characterize the effect of many NPIs (22, 23).

Another method is to use location history data enabled by the Global Positioning System (GPS) information of anonymized mobile phones, which provides accurate location on the meter scale. If provided frequently enough, one can capture colocations of pairs of devices, depending on their actual distance. An advantage compared to BLE contact tracing is that BLE relies on a person’s proximity, while the virus may also be transmitted minutes after the infected person has left (24). As a drawback of GPS-based contact analysis it should be noted that with BLE, signal strength is reduced by obstructions such as walls and can detect whether proximity is not a personal contact.

In this study, we analyze encounters or “contacts” derived from GPS information from cell phones. We employ data collected from a panel of around 1.2 million users in Germany that have opted in to provide the data for research purposes. The data records are deidentified. After estimating the aggregate daily contact distribution in a geographical region (at the level of counties), we drop all information of geolocation of all contact events. Thus, individuals cannot be identified for instance by places of frequent contacts.

With this method we are able to study the social-distancing behavior of the German population statistically. Employing the data from the panel, we can find many of the contacts between members of the panels. We are missing the majority of contacts in the entire population (those contacts where at least one person is not having a cell phone or a participating app is not installed or active). However, we argue that by using graph sampling theory we can calculate statistics of the contact number distribution for the population. We can then show that within the data there is a strong association of cell phone-derived contact data with case numbers over all phases of the epidemic.

## Method

**Fig. 1.**
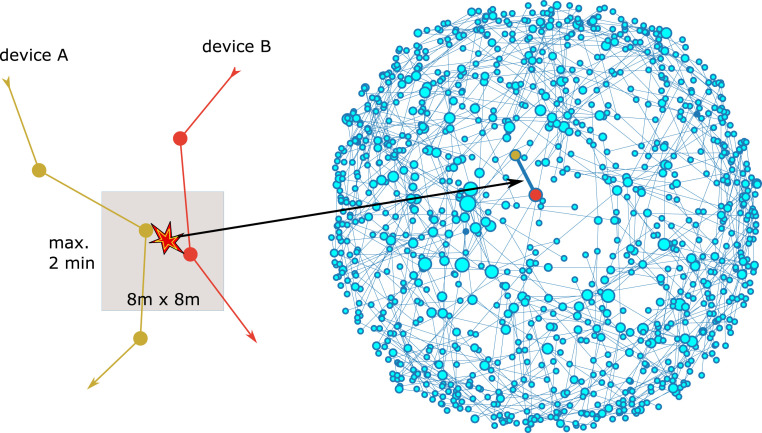
(*A*) Our method of identifying encounters by GPS coordinates rests on the colocation of two devices on a geolocation tile within a time interval of 2 min. In the contact graph, pairs of devices are linked if a cospace–time location was found at least once per day. The exemplary network shown in *B* is only the part of our total network of phones that is located in Leipzig on 29 February 2020. The size of the dots corresponds to the degree, or number of contacts, of the node. The layout of nodes is obtained from a spring-force algorithm (25).

For our analysis we assume that each cell phone is used always by the same individual. For each cell phone in our representative and anonymous panel of more than 1 million devices we obtain records that contain up to several hundred messages per day and per device. We then project the positions for each message to a predefined tile of about 8 × 8 m. Using an identification number of the tile, we then scan for coincident presence of two different individuals on the same tile with the same time stamp rounded to 2 min (Fig. 1*A* and *SI Appendix*) (see ref. 26 for a similar method). We define as a unique contact each pair of individuals that encountered one another one or more times during 1 d (27). This choice was motivated by reasoning that colocation with the same persons at home or work for long times during a day does presumably not increase the chance of infection beyond a significant limit. The collected data cover about 1% of the German population over the entire period of the pandemic, including weeks before its beginning.

For a detailed analysis of the data we use methods from complex network science. We consider a graph of contacts for each day by assigning each device to a node of the network, while each contact defines an edge between the respective nodes (Fig. 1*B*). We then focus on the graph parameters that determine the simplest measures for epidemic modeling: the mean degree and the heterogeneity of the degree distribution. For a statistical estimation of the moments of the degree distribution for the underlying “real” Germany-wide network we use graph sampling theory described in *SI Appendix*.

## Results

**Fig. 2.**
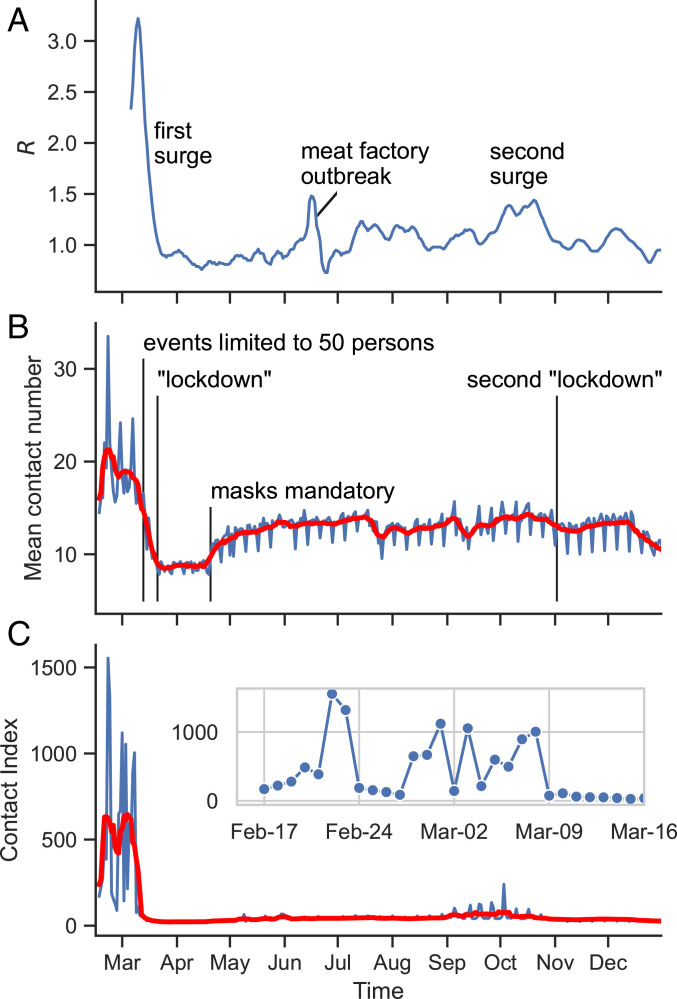
(*A*) The effective reproduction number R for Germany based on a summation of case numbers for 7 d. (*B*) Mean number of contacts per day from cell phone records (blue) and 7-d moving average (red). (*C*) Contact index per day (blue) and 7-d moving average (red).

In Fig. 2 we compare the evolution of the effective reproduction number (Fig. 2*A*) estimated from the Nowcasting case numbers for Germany [(7-d average, 4-d generation time)-R, value from ref. 28, technical details in ref. 29] with the evolution of the two considered graph parameters. Fig. 2*B* shows that the mean number of contacts of individuals per day was visibly reduced in the middle of March. This holds particularly in the initial phase of the outbreak after which the weekly averaged number of contacts per person fell from around 20 to around 9 (27 March). However, beginning in the middle of April the number of contacts increases again and reaches a relatively stable plateau of about 75% of the preoutbreak level for the rest of the time. Interestingly, the contact number increases with the beginning of mask use, prompting us to wonder whether the perceived additional safety of the masks leads to a relaxation of personal distance.

The number of contacts does not sufficiently reflect and anticipate the subsequent evolution of the incidence-based R. Our reasoning is this: While the two curves, for R and the mean contact number, roughly correspond, we note that the R value decreases synchronously with the mean degree until both hit minimal levels, which is not intuitive since the contacts should precede the infection for a few days (*SI Appendix*, Fig. S1). As well, the substantial increase of the number of contacts after April is not accompanied by an equally strong increase in R. While the number of contacts goes back up to 75% of its maximal value, R stays at values around 1.0 throughout most of the summer months. We thus conclude that the number of contacts does not sufficiently reflect and anticipate the subsequent evolution of the incidence-based R. To achieve this, a more complex metric is needed.

### Friendship Paradox and Contact Index.

**Fig. 3.**
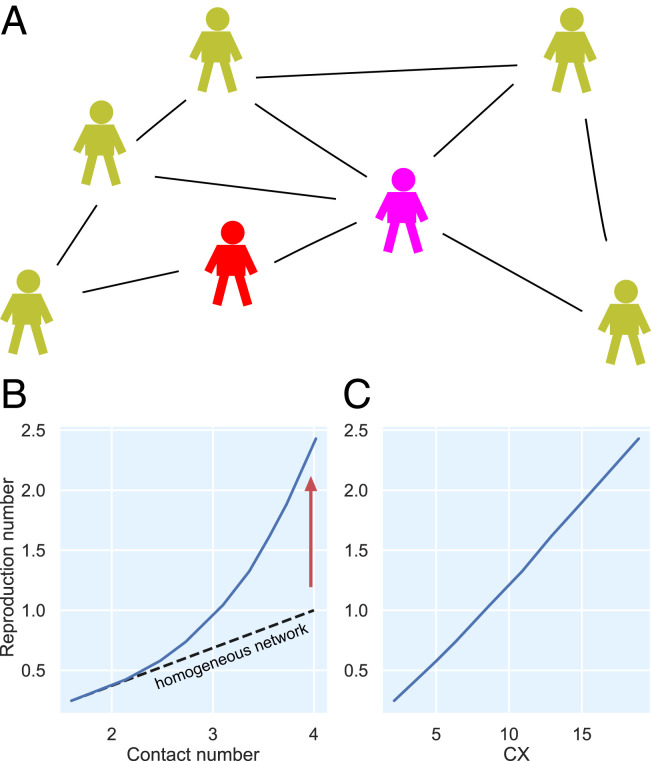
Illustration of the friendship paradox in heterogeneous contact networks. (*A*) An infected individual (red) has the same probability of giving the infection to each of the susceptible contacts. However, individuals with large number of contacts, such as the purple one, have a higher chance to be connected to the infected person. Therefore, the infection is given with a higher chance to those that have high contact number. As a result, the mean number of contacts (*B*) does not represent the effective R (blue curves) value linearly. Instead, the contact index CX (*C*) is needed to correct for the broadness of contact number distribution so that the expected linear relation is obtained. Blue curves in *B* and *C* are obtained by simulations of the SEIR model on graphs with variable degree distribution (*SI Appendix*).

It is known that for strongly heterogeneous networks, i.e., if the number of contacts is variable, the reproduction number R as defined from the links on graphs generally follows the ratio of second to first moment of the degree distribution but not the first moment (or mean contact number) (30, 31). This is a consequence of what is sometimes called the friendship paradox and can be understood as follows (Fig. 3*A*): The reproduction number includes as a factor the average number of contacts. The mean number is the first moment or $\sum_{k}kP\left(k\right)$, where P(k) is the probability distribution of the number of contacts. The mean k of the nodes that are connected to a random infected node, however, must be weighted with a further factor k since nodes with higher k are connected to the given nodes with higher probability. The mean is therefore $\sum_{k}k^{2}P\left(k\right)$ divided by a normalization $\sum_{k}kP\left(k\right)$. This ratio of second to first moment defines the (contact-based) reproduction number for networks based on the so-called configuration models. In the following, to distinguish the incidence-based R from the contact-based number, we call this number the “contact index” (CX) and compare it to R derived from the incidence numbers. For illustration we show in Fig. 3*B* an exemplary simulation of a susceptible-exposed-infectious-recovered (SEIR) model on a graph. If the graph is heterogeneous, R (blue) grows superlinear with the contact number so that the contact number underestimates R. In contrast, CX exhibits a linear association in the configuration model (Fig. 3*C*) (see *SI Appendix* for more details on the numerical simulations).

The degree distribution can indeed be very broad in the case of social networks and the moments ratio gives a very different number than the mean (the numbers are equal if the contacts are homogeneous). Particularly we find this to be the case in our contact graph indicating a strong role of heterogeneity in the contact behavior (see below and *SI Appendix*, Fig. S2).

In Fig. 2*C* the curves show the evolution of the contact index (blue) and its 7-d moving average (red). After the first lockdown, the contact index stays relatively constant and remains low even after relaxation of lockdown and during the summer. This suggests that it reflects and anticipates better the evolution of R than the mean number of contacts. Fig. 2 *C*, *Inset* shows the detailed evolution of the CX before the first lockdown and gives an idea of how the “normal” social behavior of the population is reflected in the CX. First, a strongly increased CX, up to 1,000 and more, is seen during weekends, particularly on Fridays and Saturdays. On other days of the week the CX is at a few hundred. There are notable exceptions during the working days such as 3 March, a Tuesday. On this day we identified a soccer game of the German cup to be largely responsible for the increase. The largest values on 22 and 23 February coincide with the carnival festivities during that weekend.

**Fig. 4.**
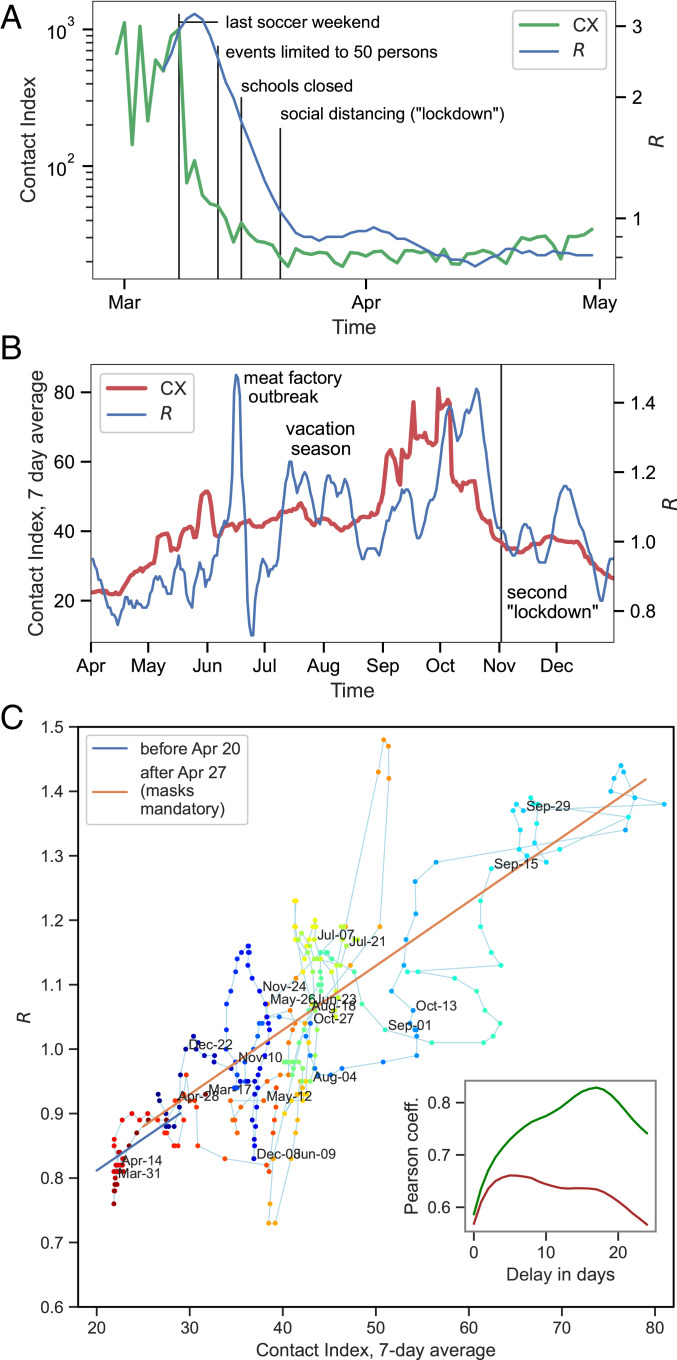
(*A*) The evolution of CX and R during and after the first surge in Germany in 2020. We have added time points of notable political interventions (vertical lines). (*B*) Evolution after the first wave shows a substantial and related increase of R and contact index. (*C*) R and the contact index exhibit an almost linear relation if plotted with a shift of 17 d. Colors denote different weeks as indicated. *Inset* shows the Pearson correlation coefficient against the number of days in delay: R versus CX in green, R versus mean k in red.

The evolution of the contact index during the first wave and its relation to the reproduction number can be further inspected in Fig. 4*A*. Indeed, we can see that strong decreases followed a number of political interventions: 1) cancellation of soccer games (three games in the first division on 8 March and one final game on 9 March) and other large public gatherings; 2) limiting of public concerts, etc., to a maximum of 50 persons (13 March); 3) closing of school (16 March); and 4) social-distancing measures (“lockdown,” 23 March). It should, however, be kept in mind that companies shifted to home office during this time. The specific effect of the various measures is thus hard to distinguish from self-imposed social distancing. Finally, we note that the CX reflects the return of R toward 1 more accurately than the total number of contacts. The CX anticipates the R decrease by about 1 wk during the first wave, while, as described above, the contact number falls concurrently with R.

The contact index also shows a strong association with the outbreak’s evolution in the phase after the first wave and during the second wave. Fig. 4*B* zooms in on the time after the first wave, exhibiting an associated of R and CX. Generally, the CX anticipates the infection numbers. This holds particularly for the onset and decay of the second surge in October, which is anticipated by a strong increase and decay of the CX. A notable omission from this coevolution is the large but short effect of a local outbreak in June (with 1,413 infections) (32) as well as an increase of R for a few weeks in July and August. We assume that a part of these infections was due to travelers who returned from vacation in other countries with higher incidence rates. Besides, base levels in Germany were low during the summer so that even a modest local outbreak can strongly affect the reproduction number. We can therefore not expect that a similar behavior is seen in the CX that reflects only connectivity within the country.

### Linear Regression of CX with *R*.

The correlation of CX and R can be assessed from Fig. 4*C*, which plots the R values versus the contact index at 17 d earlier showing the predictive power of the CX. We calculated the Pearson coefficient using weights from the uncertainty of R (*SI Appendix*). The Pearson correlation coefficient is maximized for a delay of 17 d between the CX and R with a coefficient of 0.83 (Fig. 4*C*, *Inset*, 95% confidence interval 0.79 to 0.86). A much smaller coefficient is obtained for the correlation of R and the mean contact number (red curve in Fig. 4*C*, *Inset*).

To address the possibility of spurious correlations between time series (33) we used an autoregressive–moving-average (ARMA) model to filter out autocorrelations. We found significant cross-correlations between CX and R for time lags of 14 to 16 d (see *SI Appendix* for more information). Since it is known that the optimal time lag is shifted to higher values if autocorrelations are included, a lag of 17 d is fully compatible to this result for the unfiltered time series (34).

A linear regression of R on CX for a delay of 17 d was applied for two phases: one before the mandatory use of masks (blue diagonal line in Fig. 4*C*) and one after masks were in widespread use (red diagonal line). We did not find a substantial difference in the linear approximation. Thus, in contrast to studies that find a large protective effect of mask wearing (35) (but also see refs. 36 and 37), our data do not provide significant evidence for an effect of masks in limiting of infection behavior. Other changes in health policy measures and restrictions during the same time may have confounded the effect of masks in our data. It should also be noted that a recent study found the effect of masks to be relatively small initially and to become gradually larger over several weeks (37), which would make it difficult to detect the change in our data.

The linear regression fit crosses the R = 1 threshold at a contact index of ${CX}_{crit}\approx 38$ (95% confidence interval 19 to 57). A larger CX value would drive the infection behavior into a sustained supercritical regime with R>1. This observation may be useful for the tuning of social-distancing measures and the timely assessment of the measures.

It is instructive to inspect the origin of the large CXs before the lockdown and of the CX reduction after a lockdown. We found that indeed a very broad distribution of contact numbers for individuals is responsible for the large CX values before the lockdown. After each of the two described surges (March and November), this distribution becomes much narrower, leading to a smaller CX. The contact index approach thus covers superspreader events, however, from a different point of view. In fact, a superspreader event connects many people in one place at one time. These persons might, as to the level that they are included in our mobile phone panel, pick up several additional contacts during this event and become what one could call “supercontacters.” As such, they enter the CX with a higher weight and the superspreader event is, in this sense, represented in our metric. In our opinion the notion of a superspreader event is somewhat misleading since it does not matter whether a person has many contacts and spreads the virus in one place or visits many different places during a day and spreads the virus several times.

The dependence on “supercontacting” of CX and its higher R association compared to the mean contact number is further evidence for the central role of superspreading for the infection behavior and for effective interventions (see *SI Appendix* for more details). Clearly, by far the highest effect on CX was the closing of event venues and cancellation of sport and other events in early March 2020. We expect that further research into CX and other (local and global) contact graph metrics can help to rank NPIs according to their reduction of the CX. All metrics that are used for NPI assessment have their error sources and it seems to us that, because of the early time point and technical methodology of the GPS-based contact estimation, CX can be regarded as one that is less confounded by oftentimes variable human behavior (e.g., variations in the number of tests).

## Discussion

In this paper we have devised a graph-theory–based method that can take full advantage of the information contained in bulk cell phone data. We showed a strong association between the effective R and the GPS-assessed contact behavior of the German population. We found a high correlation of R with the contact index CX, which accounts for a heterogeneous contact behavior of the population and superspreading behavior. The CX allows us to quantitatively evaluate risks for certain behavior, for instance by regional analysis (*SI Appendix*, Fig. S4), and to assess the efficacy of vaccination in reducing the CX (*SI Appendix*, Fig. S5).

It should be clear from our discussion that aggregated mobility data alone do not suffice to derive strong and reliable assertions to predict infection behavior. Aggregated mobility does not necessarily correlate with contact/transmission behavior. For instance, correlation was shown to be large directly after the first wave in the United States but much less so in the period thereafter (23). The contact data we analyze, however, do causally precede, on the population level, the infection numbers. Contact data, however, carry a higher need for ethical consideration since the geographical resolution must be high and the data can be aggregated only after the initial contact analysis. On the other hand, the high predictive power shown here and the need for data-enabled decision making may shift the political debate in the future so that more countries decide to opt for an efficient contact tracking, particularly in the statistical usage proposed by us, that can be done with deidentified data. Regardless, to make the method even safer we propose to increase data security by additional measures, such as secured storage and possible double encryptions of geolocations (26).

How plausible is the time delay of 17 d between the effective R and the contact index? Since we have used the R value based on 7-d intervals (attributed to the last day of each 14-d interval) and a 4-d average of the case numbers (attributed to the last day of the 4 d) we expect a delay of 7 + 2 d between the actual onset of symptoms and report in the table we have used (38). Thus, the delay of 17 d is close to the expected one if one adds 4 to 7 d of incubation time (3). The remaining mismatch could result from inaccuracies in the imputation of data where the onset of symptoms is not known or where no symptoms were reported (38).

The method we have presented here is not complete and may be improved in several ways in the future. First, we did not include international travel activity. During the year 2020 it was apparent that transmission of the virus between different countries strongly affects the dynamics regionally. For instance, it is was shown that almost 50% of cases in Germany during the main vacation time in July and August were related to returns from other countries (39). Further, we record for each device only the number of different phones per day in contact. That is, we do not consider whether the contact to a specific device/individual was long or short, which may play a decisive role in the transmission of the virus. Thus, we may overestimate the role of short contacts, for instance in shops. However, in view of the discussion on spreading by aerosol particles, it is possible that even a short presence of an infectious individual may be sufficient in some circumstances (24). We further note that smart phones and the specific apps the data gathering is based on are not used with any groups in the population in the same way. For instance, since cell phones are not used as frequently by children as by adults, we do not expect to cover children with our method.

It should be noted that the degree distribution is only one graph-based factor that enters the epidemic threshold. A real network could also have, for example, clustering and degree correlations that may affect the R value (31). This may also explain why the CX-R relation in our chart does not go through the origin and varies over a much wider relative range than the 7-d R value for the first wave in Germany. Further work is needed to clarify the impact of these graph properties.

## Supplementary Material

Supplementary File

## Data Availability

Some study data are available upon request.
