# Supplementary material for: Predicting the SARS-CoV-2 effective reproduction number using bulk contact data from mobile phones
Source: Proc Natl Acad Sci U S A. 2021 Jul 14;118(31):e2026731118. doi: 10.1073/pnas.2026731118 (PMC8346907; doi:10.1073/pnas.2026731118)
Supplement: Supplementary File [file pnas.2026731118.sapp.pdf]

PNAS
www.pnas.org

Supplementary Information for

Predicting the SARS-CoV-2 effective reproduction number using bulk proximity data from mobile phones, Supplementary Material

Sten Rüdiger, Stefan Konigorski, Alexander Rakowski, Jonathan Antonio Edelman, Detlef Zernick, Alexander Thieme, Christoph Lippert

Sten Rüdiger

This PDF file includes:

Figs. S1 to S12 (not allowed for Brief Reports)
Table S1 (not allowed for Brief Reports)
SI References

1. Additional analysis

A. Evolution of mean contact number. The evolution of the mean contact number k only partially reflects the succeeding evolution of the reproduction number R. This is visually evident from the figure S1. The rapid decline after the first wave occurs concurrently for k and R. As noted in the main text, we can see a strong increase of k beginning from May which is not accompanied in an increase in R. The value of CX, however, stays roughly constant during this time, explaining the constant value of R (see main text).

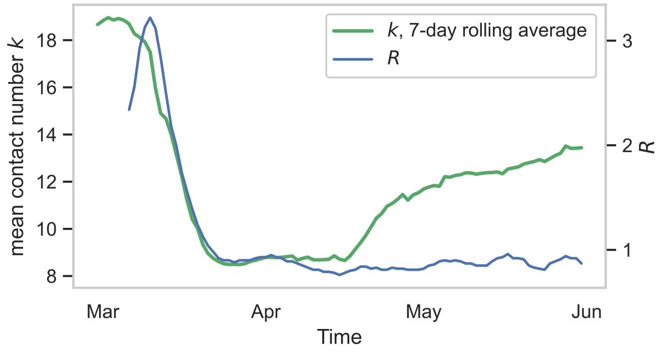

Fig. S1. The initial surge in March 2020 in Germany ended with a strong decline of the mean contact and the R value over the month March. The evolution does not fit the naive expectation that a change in k is followed by that of R with a time lag of at least a few days. As well there is a noticeable increase in k from April to June which is not reflected in the R evolution.

B. Distribution of degrees. Evaluating the number of contacts per device we find that the degree of nodes, i.e., the number of contacts of each person, is broadly distributed with a long tail before the lockdown (Fig. S2, blue line) while at later dates after the first wave, the distribution has a much shorter tail and is highly concentrated around few contacts (compare brown and green lines). Thus initially there are many individuals with large numbers of contacts who would be potential 'super-spreaders' (called hubs in network theory), but the lockdown clearly led to a reduction of the number of such individuals at later weeks. The same pattern is observed before and after the second wave and lockdown (red and purple dots).

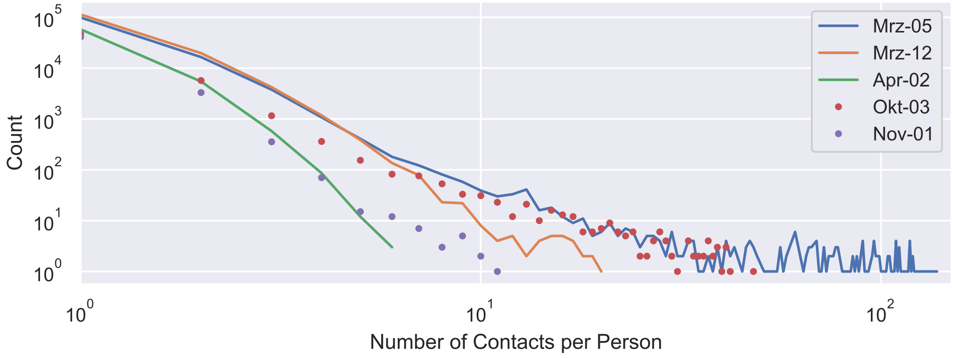

Fig. S2. Histogram of number of contacts per person on three days before and after the first wave (solid lines) and two days before and during the second wave (dots): March 5, March 12 before official lockdown, March 19 (histogram cut off at 30 contacts) after ban of large gatherings and local regulations closing restaurant and other public venues. October 3 was a Saturday and also the national holiday with public gatherings. November 1 was one day before the second lockdown.

C. Regional mapping of CX. The mobile phone data used by us can also be mapped for a regional analysis. For this investigation we estimated a home county for each individual by selecting the county with the most samples from a device in a given week. Then the number of contacts per day for this device is assigned to this county. We can see the usefulness of the local analysis in Fig. S3 in that the highest CX values occurred in the cities that held soccer games during March, 1st 2020.

Investigating the contact index for large cities we find in general much higher values, which complements the observation that large cities had an earlier onset of the epidemic. In Fig. S4 the five cities Hamburg, Cologne, Stuttgart, Munich and Frankfurt am Main are shown in yellow whereas the counties with the lowest number of inhabitants (<100,000) are shown in pink. The city with the peak value at around CX = 1,200 is the city of Cologne and the week of large CX values is the one around carnival.

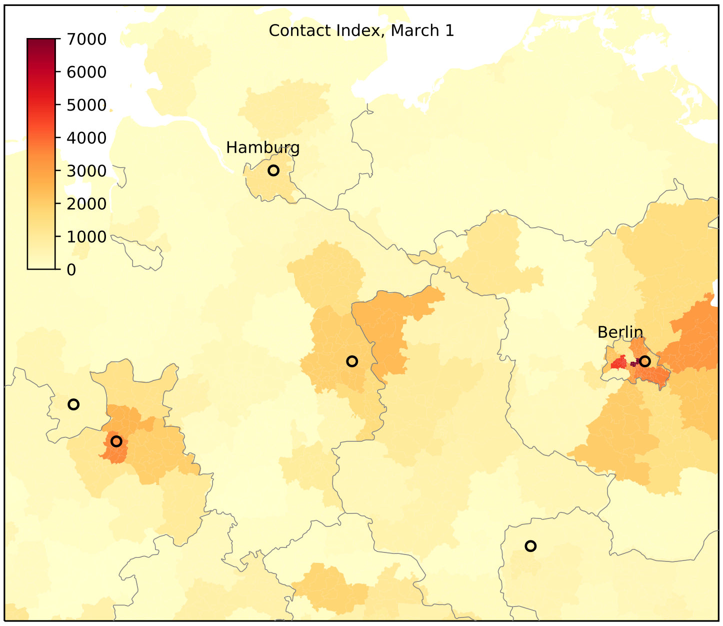

Fig. S3. The CX can be locally calculated by only evaluating cell phones located mostly in a specific county (while keeping all contacts, as well outside of the county, of that cell phone for the calculation). The map shows the CX on March 1, 2020, a Sunday. Several professional soccer matches were held on that day. The circles show cities that took part in the games from the two top tiers.

D. Vaccination campaign. In the light of the network hubs that are present in the contact graph for March 12-19, we can discuss implications of our findings for vaccination strategies. This seems particularly interesting considering that at least for the initial stages of a vaccination only a small share of the population can be immunized. It is widely assumed that for a herd immunity a share of the population of 60 % or more must be vaccinated (1). However, this threshold assumes "well-mixedness" of the population while for outbreaks on graphs the heterogeneity of contacts must be taken into account. In Fig. S5 we analyse a random model. We base the calculation on the distribution of degrees in our sampled network for the week March 12-19, 2020. For the random strategy we uniformly sample and remove (i.e., vaccinate) nodes of all degrees k with the same probability. We judge the strategy using our threshold of $\mathrm{CX}_{\mathrm{crit}} = 38$ which corresponds to an R of 1.0. In order to reduce CX below the critical value, starting from the social situation in early March, we need a vaccination fraction of about 95 %. It is conceivable that by targeting nodes with large numbers of connections (i.e., hubs) one can reduce the fraction of nodes that need to be immunized.

The calculation of CX for the network with immunization proceeds in the same way as for the sampled networks considered before. In the synthetic experiment nodes are removed randomly from the sequence of degrees of the sampled network. Then the first and second moments are calculated from the distribution of the remaining degrees. Then CX of the entire population is obtained as described below as $N_{\mathrm{tot}}/N_{\mathrm{obs}}(\langle k^2\rangle_{\mathrm{vacc}}/\langle k\rangle_{\mathrm{vacc}} - 1)$, where N_{tot} is the number of inhabitants and N_{obs} is the number of cell phones in the panel.

A theoretical estimate of the vaccination CX is obtained in a similar way by starting from the CX of the complete sampled network (726 for the week considered here) and employing the vaccination share as additional factor reducing the number of nodes: $(1-v)N_{\mathrm{tot}}/N_{\mathrm{obs}}(\langle k^2\rangle_{\mathrm{vacc}}/\langle k\rangle_{\mathrm{vacc}} - 1)$, where v is the share of the vaccinated population. This estimate fits well to the results from the synthetic experiment, Fig. S5.

2. Theory and Methods

In this Appendix, we describe our specially designed algorithm that identifies "contacts" from the traces based on the following rationale: If GPS pings arrive from two distinct cell phones that are close in space and time, then we denote this event as a

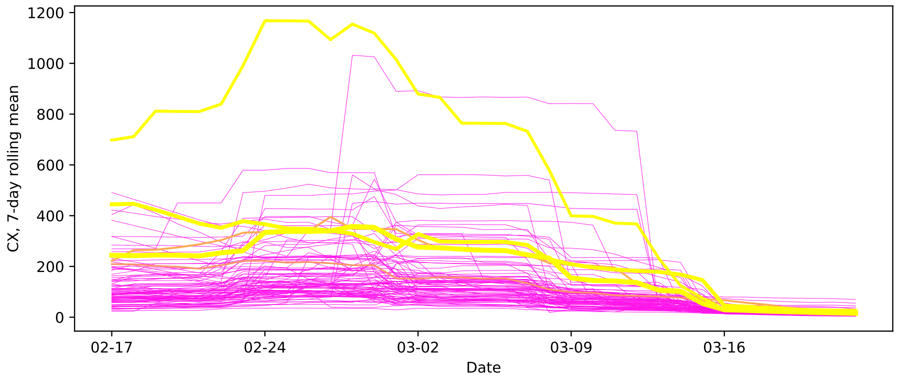

Fig. S4. Comparison of CX of five of the largest German cities (yellow) and the smallest counties in Germany showing that cities have comparatively large CX values.

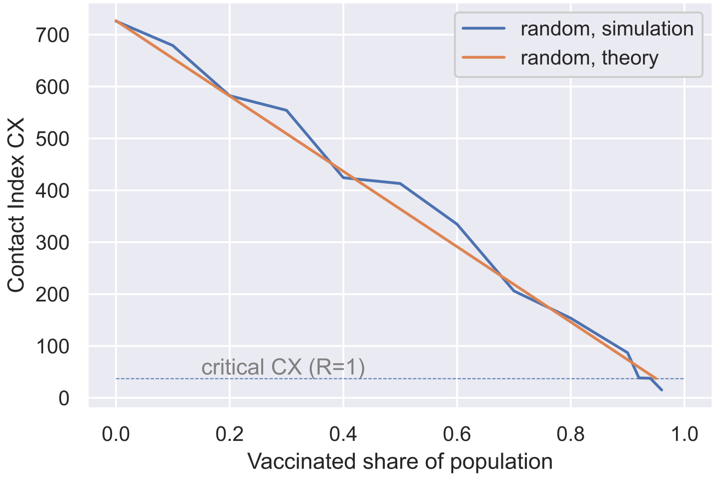

Fig. S5. Contact Index for random vaccination strategies where a certain share v of the population has been immunized. The computation is based on the contact graph for March 12-19. We judge the efficacy of the campaign by comparing the CX to the critical value (horizontal line). Herd immunity is achieved at a level of 95 % vaccination. This behavior is expected for networks with a strong heterogeneity in the contact numbers (2).

"contact" and use it as a proxy for a human physical contact.

Our data allows estimating the number of real-world contacts for the entire population of Germany. However, a large part of these real contacts is missing from our cell phone sampling for two main reasons: (A) We only cover a fraction of devices. (B) We only cover times when the cell phone is sending a ping. In more detail regarding (A), we cover about 800,000 GPS-enabled devices per day, so that the majority of contacts for an individual goes undetected. As there are about 83 million people in Germany, we can expect to cover about 1% of the population. Regarding (B), a typical cellphone sends about 200 pings per day. In order to cover the entire day, one ping every two minutes i.e. 720 pings per day are needed. Thus, only about 28% of the time of the day is covered for the average device. Assuming for simplification that the time of pings are independent for different devices, a lower bound estimate of the probability that a contact between two devices was observed is $0.28 \times 0.28 \approx 0.1$. So, according to this rough calculation, we can expect to track at least $0.01^2 \times 0.1 = 0.001\%$ of all contacts between any two people living in Germany.

The average number of cell phones registered during a day was about 800,000. Per day we find between 20,000 and 160,000 devices that had at least one match. The total number of matched pairs varied between 150,000 (before lockdown) and 12,000 (during lockdown) per day.

A. Sampling of nodes. We now describe more formally how measures of the sampled network, such as mean contacts and second moment of contacts, relate to the respective measures of the original full contact network for all cell phones. We focus on sampling of devices described in restriction (A) and ignore restriction (B) for simplification, since it is similar to the sampling of nodes in restriction (A) and would just require a re-scaling of the parameter p in equation (14).

In the following, let G denote the full network or graph of all cell phones and let M denote the maximal degree of a node in G. As a reminder, the degree of a node (i.e., person) equals the number of contacts of this person. Following Zhang et al. (3) we let N denote the vector containing the degree counts of the nodes (an alternative way of deriving our results is based

on the Horvitz-Thompson estimate (4, 5)). N has length $M+1$ and the k-th entry of N contains the number of nodes that have degree k, i.e. the number of devices that have k contacts. Thus, N contains the counts of the number of cell phones having k links (contacts) to other cell phones.

In the sampling of phones according to (A), we assume that each phone is sampled from G with the same probability p, resulting in the sampled graph G^*. This situation is also described as *induced network sampling* in network theory (3). The induced network G^* includes all sampled nodes as well as all links from G that connect the sampled nodes in G^*.

The vector of the expected values of the degree counts of the sampled network, N^*, is $E(N^*) = PN$, Here, P is a matrix of entries $P(k,k')$ that describe the probability that a node of degree k' in G is selected and has degree k in G^*. For induced sampling, P is:

$$P_{\text{ind}}(k,k') = \begin{cases} \binom{k'}{k} p^{k+1}(1-p)^{k'-k} & \text{for } 0 \le k \le k' \le M, \\ 0 & \text{for } 0 \le k' < k \le M. \end{cases} \quad [1]$$

Thus the k-th entry $E(N^*(k)) = \sum_{k',k\le k'} N(k')\binom{k'}{k}p^{k+1}(1-p)^{k'-k}$.

In the following we assume that the particular sampling given by our mobile phone records gives rise to a N^*_{ind}, which can be approximated by $E(N^*)$ for large networks, from which we can calculate the degree moments for the original network.

B. Derivation of the contact index CX. Let $\langle k\rangle$ denote the mean degree of nodes in G: $\langle k\rangle = \sum_{k=0}^{M} kN(k)/(\sum_{k=0}^{M} N(k))$. We first show that the mean $\langle k\rangle_{\text{ind}}$ of the sampled graph is linearly related to the mean of the original graph:

$$\langle k\rangle_{\text{ind}} \approx \frac{\sum_{k=0}^{M^*} kE(N^*(k))}{\sum_{k=0}^{M^*} E(N^*(k))} \quad [2]$$

$$= \frac{\sum_{k,k',k\le k'} kN(k')\binom{k'}{k}p^{k+1}(1-p)^{k'-k}}{\sum_{k,k',k\le k'} N(k')\binom{k'}{k}p^{k+1}(1-p)^{k'-k}} \quad [3]$$

$$= \frac{p\sum_{k'} N(k')\sum_{k,k\le k'} k\binom{k'}{k}p^{k}(1-p)^{k'-k}}{p\sum_{k'} N(k')\sum_{k,k\le k'} \binom{k'}{k}p^{k}(1-p)^{k'-k}} \quad [4]$$

$$= \frac{p^2\sum_{k'} k'N(k')}{p\sum_{k'} N(k')} \quad [5]$$

$$= p\langle k\rangle \quad [6]$$

The equality of (4) and (5) follows since $\sum_{k,k\le k'} k\binom{k'}{k}p^k(1-p)^{k'-k}$ is the mean value of the binomial distribution $B(k',p)$ which equals $k'p$, and $\sum_{k,k\le k'}\binom{k'}{k}p^k(1-p)^{k'-k}$ is the sum of all probabilities in $B(k',p)$ which is 1. Similarly, we find for the second moment:

$$\langle k^2\rangle_{\text{ind}} \approx \frac{\sum_k k^2E(N^*(k))}{\sum_k E(N^*(k))} \quad [7]$$

$$= \frac{\sum_{k,k',k\le k'} k^2N(k')\binom{k'}{k}p^{k+1}(1-p)^{k'-k}}{\sum_{k,k',k\le k'} N(k')\binom{k'}{k}p^{k+1}(1-p)^{k'-k}} \quad [8]$$

$$= \frac{p\sum_{k'} N(k')\sum_{k,k\le k'} k^2\binom{k'}{k}p^{k}(1-p)^{k'-k}}{p\sum_{k'} N(k')\sum_{k,k\le k'} \binom{k'}{k}p^{k}(1-p)^{k'-k}} \quad [9]$$

$$= \frac{p\sum_{k'}(k'(k'-1)p^2+k'p)N(k')}{p\sum_{k'} N(k')} \quad [10]$$

$$= p^2\langle k^2\rangle - (p^2-p)\langle k\rangle \quad [11]$$

Here, (7) is the definition of the second moment, (10) follows from (9) since the second moment for the binomial distribution $B(k',p)$ is $p^2k'^2 + k'(p-p^2)$ and $\sum_{k,k\le k'}\binom{k'}{k}p^k(1-p)^{k'-k} = 1$, and (11) follows from (10) because of the definitions of the first and second moments of $N(k')$. Finally, we describe how the ratio $\langle k^2\rangle/\langle k\rangle$ of the original graph can be obtained from the sampled graph via $\langle k\rangle_{\text{ind}}$ and $\langle k^2\rangle_{\text{ind}}$:

$$\frac{\langle k^2\rangle}{\langle k\rangle} \approx \frac{\frac{1}{p^2}\left(\langle k^2\rangle_{\text{ind}} - (p-p^2)\langle k\rangle\right)}{\langle k\rangle} \quad [12]$$

$$= \frac{\langle k^2\rangle_{\text{ind}}}{p\langle k\rangle_{\text{ind}}} - \left(\frac{1}{p}-1\right) \quad [13]$$

$$= \frac{1}{p}\left(\frac{\langle k^2\rangle_{\text{ind}}}{\langle k\rangle_{\text{ind}}} - 1\right) + 1. \quad [14]$$

This ratio $\langle k^2\rangle/\langle k\rangle$ is of interest, since it describes the growth rate of an infection phase in an uncorrelated network (6). Since $\langle k^2\rangle_{\text{ind}}$ is larger or equal to $\langle k\rangle_{\text{ind}}$, (14) is non-negative and since p is small in our sampling, we can ignore the addition of the constant 1. Thus we define the contact index CX as

$$\text{CX} := \frac{N_{\text{tot}}}{N_{\text{obs}}}\left(\frac{\langle k^2\rangle_{\text{ind}}}{\langle k\rangle_{\text{ind}}} - 1\right),$$

where p has been replaced by the ratio $N_{\text{obs}}/N_{\text{tot}}$, where N_{obs} is the number of devices observed during a day and N_{tot} is the total number of devices/users in the considered area.

C. Effective R calculation. The effective reproduction number R values in our analysis have been obtained from the RKI Nowcasting website (7). We are using the seven-day averaged numbers based on a generation time of four days. For a given day d, R is calculated as the ratio of the sums of infections for days d to $d+6$ and $d-7$ to $d-1$ (7). This number is then attributed to day $d+6$.

The calculation of the case number R-values are based on various further considerations. The RKI must impute the beginning date of illness for about one-third of infected persons. Subsequent reports from local health administrations must be interpolated by the so-called Nowcasting method. In all of our analysis we use the 7-day R-value calculated by RKI, in which a rolling 7-day average is first calculated for each day before considering 4-day periods, in order to compensate for weekly fluctuations. The RKI excludes the case numbers of the last 4 days when calculating the R-values, as these are still too unreliable due to reporting delays.

An uncertainty of the R value is given in (7) by an interval. The 95% prediction intervals show the uncertainty due to the adjustment after the delay in diagnosis and reporting as well as due to the partially missing data on the onset of the disease (8). We use the inverse of the interval length for each day for the calculations of cross correlations with R for each day.

D. Statistical analysis. In the analysis, we estimate the contact index from a sample of nodes from the full network of cell phones in Germany. We present descriptive statistics and temporal trends of the number of contacts as well as of the contact index. Finally, we investigate their association with infection rates assessed by R by estimating their Pearson correlation coefficient. The Pearson correlations and their p-values were determined using Python's Scipy package, version 1.3.1.

E. GPS location data. The investigation relies on GPS location history data that is collected via a Software Development Kit (SDK) developed for the primary purposes of assessing the quality of cell phone networks. Cell phone data is collected by the SDK implemented in more than one million cell phones in Germany. Per day data was received from 1.15 to 1.4 million cell phones during March to July 2020. The legal conditions for the processing of the data were described in a report by A. Böken on May 11, 2020. Data records are anonymous. In a first step the number of contacts for each device is determined so that no positional information is retained. Then the data is aggregated by the number of devices that have a certain number of contacts. Only these aggregated numbers are used for further analysis.

F. Probability of contact detection and real distance between individuals. The epidemiology of SARS-CoV-2 indicates that most infections are caused by close contacts through respiratory droplet transmission with a short range, e.g. 1.5m to 2m (9) A feasible algorithm for contact detection from GPS data needs to have acceptable accuracy, while still providing efficacy in regards to computation time. Since GPS pings of several million individuals are assessed over a longer time span, a vast amount of data needs to be analyzed. Therefore, we map GPS positions to an 8m x 8m grid and assign a time stamp with a resolution of 2 minutes. Each tile within the grid has a unique identification number (tile ID). If entries have identical tile IDs and time stamps (which were rounded to two minutes), this is accounted as a contact. Contact detection might fail in dependence of the choice of grid placement, if GPS positions get mapped to different tiles even though they are in close proximity. This effect becomes more likely with increasing distance between GPS coordinates.

For evaluation of this algorithm, we used a numerically approach: we set a point p_1 to the origin coordinates and a point p_2 to random coordinates and distance d to p_1. We then create a tile with random positions which includes p_1. If p_2 is also within the tile, the contact with distance d was successfully detected. By repeating this algorithm, a curve for the probability of contact detection in relation to distances between GPS positions can be calculated (Fig. S6A). To calculate a similar graph for the real positions between individuals, the inaccuracy of GPS has to be taken into consideration. In order to do this, we randomly shifted p_1 and p_2 according to the known inaccuracy of GPS (mean error 0.95 m +- 1.05 m standard deviation (10) The result can be seen in Fig. S6B.

3. SEIR model simulations on networks

For the model simulations on a network of nodes shown in Fig. 3 of the main text we adopt the SEIR model where each node is in one of the four SEIR states: susceptible, exposed, infectious, and recovered. Each infected node can infect connected nodes. For the network of nodes we first choose a degree distributions and randomly link 1 million nodes according to this distribution (configuration model). We determine for each graph the mean contact number and CX. The R value is obtained from averaging 1000 simulation runs. Results from additional simulations are shown in Fig. S7.

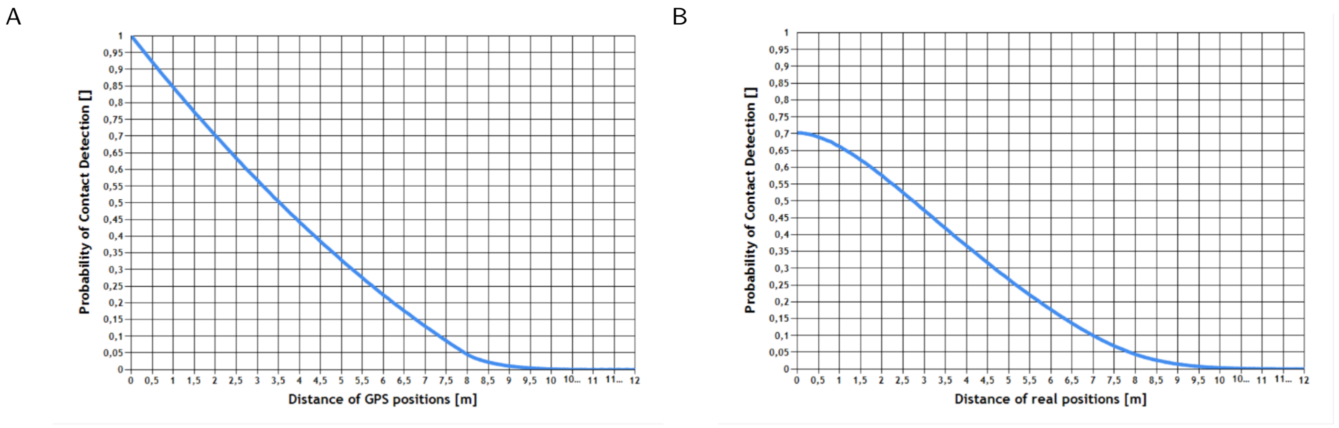

Fig. S6. Probability of contact detection. See text for a details.

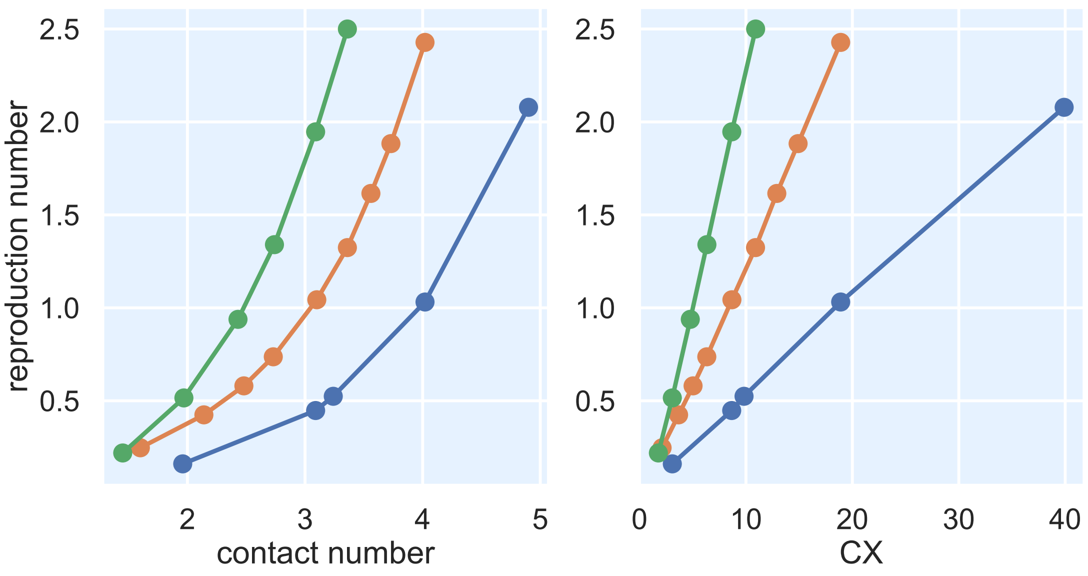

Fig. S7. Results of numerical simulations with an SEIR-model and different infectivities λ: 0.02 (blue), 0.05 (red), 0.1 (green). Other parameters: μ=0.33, χ=1.0.

A. SEIR Model of Epidemic Spreading. The SEIR model is a simple mathematical model for transmission of diseases in a population of individuals. Every individual is in either one of four SEIR states. The transitions between the states are

$$S \xrightarrow{\lambda} E \xrightarrow{\chi} I \xrightarrow{\mu} R, \qquad [15]$$

where λ and μ are the probabilities of infection and recovery per time step, respectively, and χ determines how long the individual stays in incubation.

B. SEIR on a graph. Consider now a network of nodes on which the infection spreads by specified contacts. Every node of the network is an individual. If a node is susceptible and at least one of its connected nodes is infected, it becomes infected as well with probability λ during a time step, whereas an infected node recovers with probability μ during the time step.

During each time step, for every S-node we calculate the probability λ for any connected I-node and let the S-node become infected according to this probability. If several connected nodes are infected, we randomly shuffle the order by which the transition is tested. Individuals in state R are immune and cannot be infected again. Note that for simplicity we consider λ and μ as probabilities for a discrete time step of unit length, which means that we do not obtain exponential waiting time distribution for the transitions (11).

We initially start with a network of susceptible nodes and a small number (100 to 1000 nodes) of randomly chosen infected site.

The infection numbers are added for 1000 runs, growth rates are obtained for time steps 11 to 19, averaged, and the Rs are obtained using the distribution of generation times and formula 3.6 in (12).

C. Graph generation. The degree distribution is parameterized by a number $k_{\max}$ in the following way: We first generate a probability distribution for the degrees of nodes. In a second step a graph is generated using the configuration model. The degrees are calculated from an interval from 1 to a selected number $k_{\max}$. A random number ρ in the interval 0 to 1 is chosen and projected by calculating $k_{\max}/(\rho(k_{\max}-1)+1)$ and taking the integer value of this number. The larger $k_{\max}$ the larger the mean degree and the ratio of second and first moments.

4. Panel structure

The statistics of the mobile phone user panel is shown in Table S1.

5. Autocorrelation analysis

A common problem in time series forecasting is the presence of autocorrelations in the data, rendering consecutive values not independent. This can in turn lead to spurious correlations when analyzing for cross-correlations between separate samples (13). We performed the following “prewhitening” analysis in order to asses that our findings are not incidental and indeed meaningful. It consists of:

1. Assessing the autocorrelation of the series of R values
2. If necessary, prewhitening R by fitting an autoregressive model to the data, and assessing the autocorrelation of the residuals
3. Filtering the predictor variable (CX)
4. Computing the Cross-Correlation Function (CCF) on the residuals of both R and CX to identify non-spurious cross-correlations

A. Autocorrelation in the original data. The Ljung-Box test statistic for the first lag is ≈ 214 with a p-value of less than 10^{-47}, giving strong indications for the presence of autocorrelation in the data and necessitating the prewhitening step. We also performed the Augmented Dickey-Fuller (ADF) test, with the null hypothesis of a non-stationary sample, yielding a test statistic of ≈ -2.28 with a p-value of ≈ 0.18, thus not rejecting the null. Additionally, plots of the Autocorrelation Function (ACF) and Partial Autocorrelation Function (PACF) are shown in Figure S8, revealing several significant autocorrelated lags.

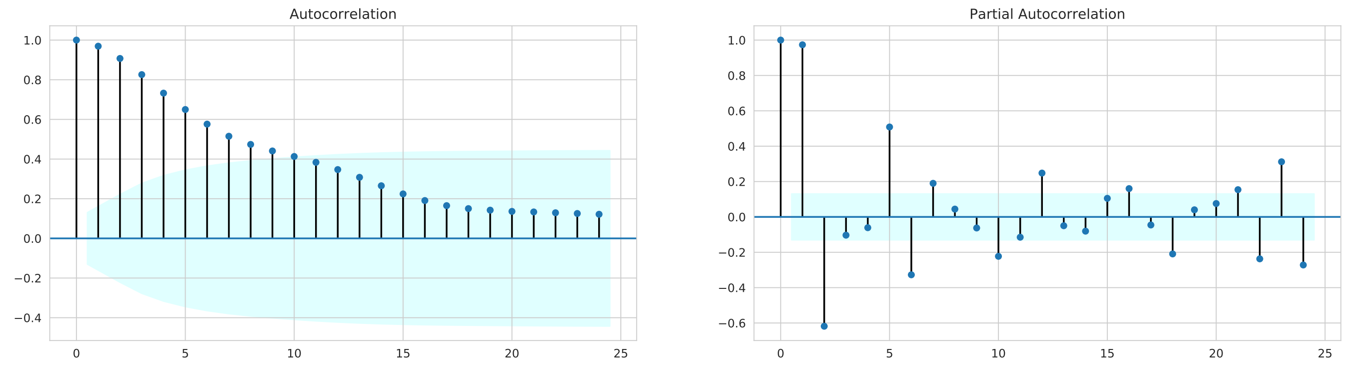

Fig. S8. The Autocorrelation (left) and Partial Autocorrelation (right) Functions for the R values, for lags up to 25 days.

B. Prewhitening. To remove the autocorrelations we fitted an autoregressive-moving-average (ARMA) model of orders $p = 1, q = 1$, where p is the order of the autoregressive and q of the moving average parts respectively. The Ljung-Box statistic for the residuals was ≈ 0.14, with a p-value of ≈ 0.71 The ADF test statistic and the corresponding p-value were ≈ -4.75 and less than 10^{-4} respectively. This was further confirmed by a visual inspection of the residuals of the model. Figure S10 shows residuals plotted as a time series, which seem to resemble a white noise process, except for the time period corresponding to the meat factory outbreak. A plot of the PDF estimate of residuals is shown on Figure S11, which resembles a zero-centered Normal distribution. The two visible outlier “bumps” are located around -1.3 and 1.5. These correspond to the residual values of the meat factory outbreak seen in Figure S10. The ACF and PACF plots for the residuals are shown in Figure S9.

C. Cross Correlation Analysis. We use the AR model fitted to the R values to additionally filter the values of CX, obtaining their residuals. We then computed the CCF on the two filtered (prewhitened) series, and plot the values of the CCF for lags 1 to 30 (days) in Figure S12. Significant values would indicate non-spurious correlations in the original data. Such correlations are indeed present, with lags 15 and 16 exceeding the 95% confidence intervals.

		basis	panel
Sex	men	50	60
	women	50	40
Age	16 - 19 years	5	4
	20 - 29 years	16	13
	30 - 39 years	16	15
	40 - 49 years	18	19
	50 - 59 years	20	24
	60 - 69 years	13	17
	70 years and older	11	9
Education	no degree	2	1
	primary school, no information	28	29
	secondary school	32	38
	high school	16	13
	university degree	21	18
Occupation	in education	10	8
	working (including temporarily unemployed)	66	67
	pensioners	17	17
	not working, no information	7	8
Number of persons in household	1 persons	21	17
	2 persons	37	46
	3 and more persons	41	37
Own net income	up to 1,000 EUR	27	25
	1,000 to 1,500 EUR	21	20
	1,500 to 2,000 EUR	16	17
	2,000 to 2,500 EUR	12	13
	2,500 to 3,000 EUR	7	7
	3,000 to 4,000 EUR	4	5
	4,000 to 5,000 EUR	2	2
	5,000 to 7,000 EUR	2	2
	7,000 EUR or more	1	2
	no income	7	7
Net income in household	up to 1,000 EUR	6	6
	1,000 to 2,000 EUR	24	24
	2,000 to 3,000 EUR	25	26
	3,000 EUR or more	44	44
Area	Germany - west	83	78
	Germany - east	17	22

Table S1. Structure of data collection panel (March 2020)

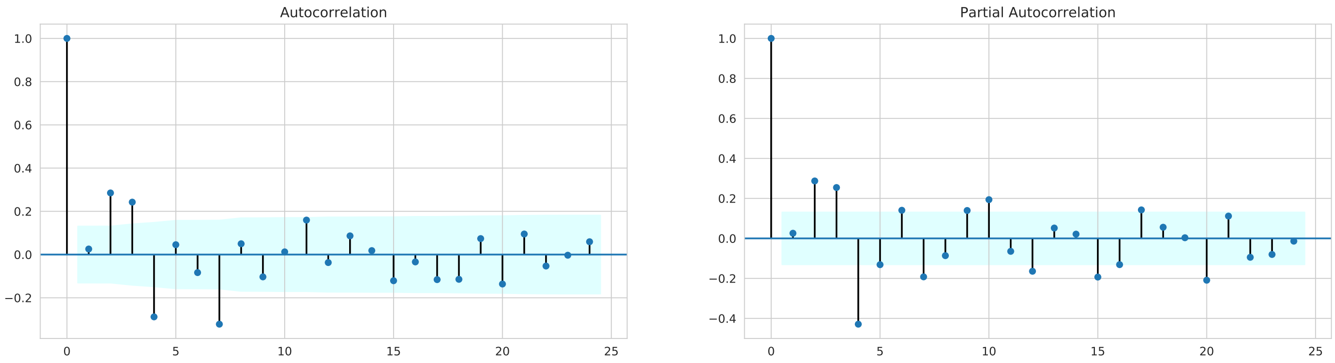

Fig. S9. The Autocorrelation (left) and Partial Autocorrelation (right) Functions for the residuals of the ARMA model fitted on R values, for lags up to 25 days.

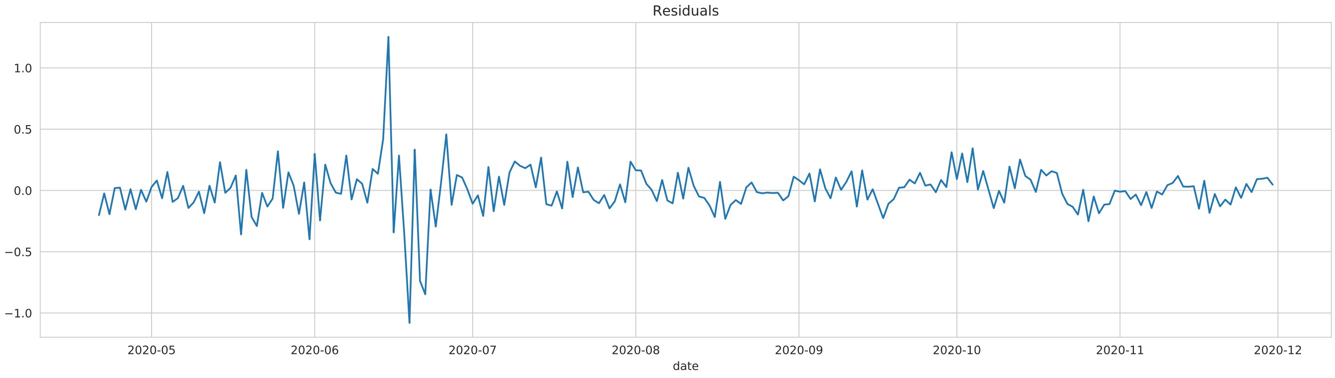

Fig. S10. Residuals of the Autoregressive model fitted to R values plotted against time.

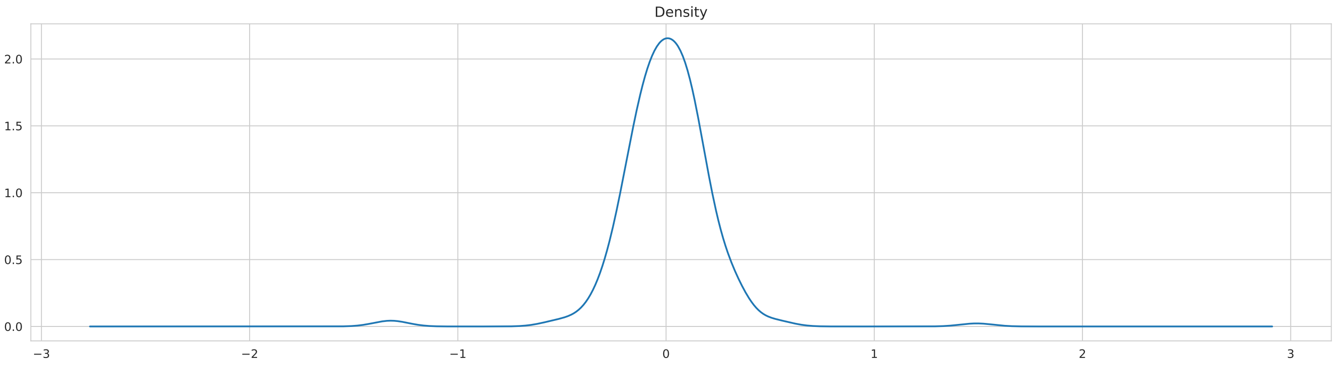

Fig. S11. Estimated probability density function of the distribution of residuals of the Autoregressive model fitted to R values.

References

1. T Britton, F Ball, P Trapman, A mathematical model reveals the influence of population heterogeneity on herd immunity to sars-cov-2. *Science* **369**, 846–849 (2020).
2. R Pastor-Satorras, A Vespignani, Immunization of complex networks. *Phys. review E* **65**, 036104 (2002).
3. Y Zhang, ED Kolaczyk, BD Spencer, , et al., Estimating network degree distributions under sampling: An inverse problem, with applications to monitoring social media networks. *The Annals Appl. Stat.* **9**, 166–199 (2015).
4. O Frank, Estimation of graph totals (1977).
5. ED Kolaczyk, Sampling and estimation in network graphs in *Statistical Analysis of Network Data.* (Springer), pp. 1–30 (2009).
6. R Pastor-Satorras, C Castellano, P Van Mieghem, A Vespignani, Epidemic processes in complex networks. *Rev. modern physics* **87**, 925 (2015).

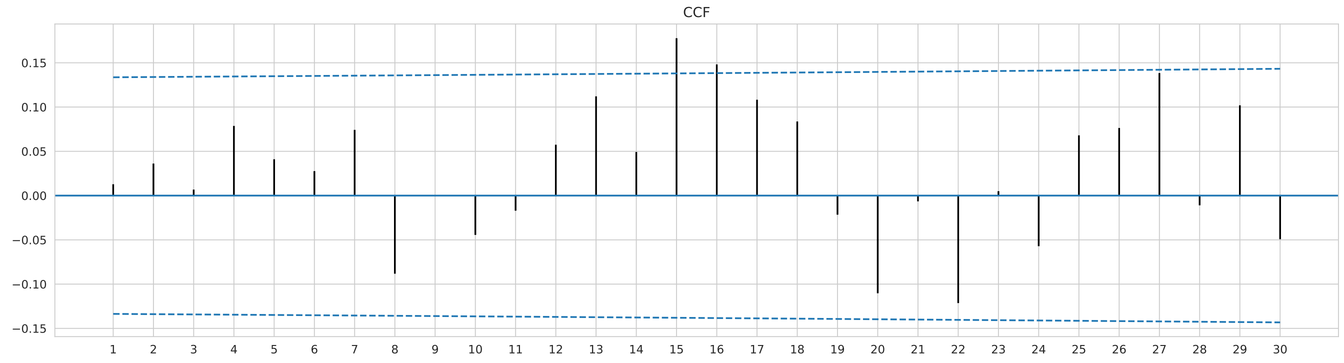

Fig. S12. Cross-correlation function between residuals of the Autoregressive model fitted to R values and filtered CX, for lags from 1 to 30 days. Dashed lines indicate the 95% confidence intervals.

7. Tabelle mit nowcasting-zahlen zur r-schätzung [internet]. (https://www.rki.de/DE/Content/InfAZ/N/Neuartiges_Coronavirus/Projekte_RKI/Nowcasting.html) (2020).
8. M an der Heiden, O Hamouda, Schätzung der aktuellen entwicklung der sars-cov-2-epidemie in deutschland–nowcasting (2020).
9. WH Organization, , et al., Report of the who-china joint mission on coronavirus disease 2019 (covid-19) 16-24 february 2020. geneva (2020).
10. Faa gps performance analysis report (2017).
11. S Rüdiger, A Plietzsch, F Sagués, IM Sokolov, J Kurths, Epidemics with mutating infectivity on small-world networks. *Sci. reports* **10**, 1–11 (2020).
12. J Wallinga, M Lipsitch, How generation intervals shape the relationship between growth rates and reproductive numbers. *Proc. Royal Soc. B: Biol. Sci.* **274**, 599–604 (2007).
13. RT Dean, WT Dunsmuir, Dangers and uses of cross-correlation in analyzing time series in perception, performance, movement, and neuroscience: The importance of constructing transfer function autoregressive models. *Behav. research methods* **48**, 783–802 (2016).
